# Saturated and mono-unsaturated lysophosphatidylcholine metabolism in tumour cells: a potential therapeutic target for preventing metastases

**DOI:** 10.1186/s12944-015-0070-x

**Published:** 2015-07-11

**Authors:** Anna Raynor, Peter Jantscheff, Thomas Ross, Martin Schlesinger, Maurice Wilde, Sina Haasis, Tim Dreckmann, Gerd Bendas, Ulrich Massing

**Affiliations:** Department of Lipids & Liposomes, Tumor Biology Center, Clinical Research, Breisacher Str. 117, 79106 Freiburg, Germany; ProQinase GmbH, Breisacher Str. 117, 79106 Freiburg, Germany; Department of Pharmaceutical Chemistry, University of Bonn, An der Immenburg 4, 53121 Bonn, Germany; Institute for Pharmaceutical Sciences, University of Freiburg, Albertstr. 25, 79104 Freiburg, Germany

**Keywords:** Lysophosphatidylcholine, LysoPC, Fatty acid, Cancer, Metastasis, Mouse metastasis model

## Abstract

**Background:**

Metastasis is the leading cause of mortality in malignant diseases. Patients with metastasis often show reduced Lysophosphatidylcholine (LysoPC) plasma levels and treatment of metastatic tumour cells with saturated LysoPC species reduced their metastatic potential *in vivo* in mouse experiments. To provide a first insight into the interplay of tumour cells and LysoPC, the interactions of ten solid epithelial tumour cell lines and six leukaemic cell lines with saturated and mono-unsaturated LysoPC species were explored.

**Methods:**

LysoPC metabolism by the different tumour cells was investigated by a combination of cell culture assays, GC and MS techniques. Functional consequences of changed membrane properties were followed microscopically by detecting lateral lipid diffusion or cellular migration. Experimental metastasis studies in mice were performed after pretreatment of B16.F10 melanoma cells with LysoPC and FFA, respectively.

**Results:**

In contrast to the leukaemic cells, all solid tumour cells show a very fast extracellular degradation of the LysoPC species to free fatty acids (FFA) and glycerophosphocholine. We provide evidence that the formerly LysoPC bound FFA were rapidly incorporated into the cellular phospholipids, thereby changing the FA-compositions accordingly. A massive increase of the neutral lipid amount was observed, inducing the formation of lipid droplets. Saturated LysoPC and to a lesser extent also mono-unsaturated LysoPC increased the cell membrane rigidity, which is assumed to alter cellular functions involved in metastasis. According to that, saturated and mono-unsaturated LysoPC as well as the respective FFA reduced the metastatic potential of B16.F10 cells in mice. Application of high doses of liposomes mainly consisting of saturated PC was shown to be a suitable way to strongly increase the plasma level of saturated LysoPC in mice.

**Conclusion:**

These data show that solid tumours display a high activity to hydrolyse LysoPC followed by a very rapid uptake of the resulting FFA; a mechanistic model is provided. In contrast to the physiological mix of LysoPC species, saturated and mono-unsaturated LysoPC alone apparently attenuate the metastatic activity of tumours and the artificial increase of saturated and mono-unsaturated LysoPC in plasma appears as novel therapeutic approach to interfere with metastasis.

## Background

Metastatic spread is the leading cause of death in the course of malignant diseases, causing about 90 % of all cancer deaths [1]. While conventional therapeutic approaches target distinct tumour cells, there is no standard therapy available which specifically interferes with the certain steps of the metastatic process. Cancer patients often show dramatically reduced phospholipid (PL) plasma levels. For example, prostate cancer patients [2] and patients with acute leukaemia [3] had significant lower levels of total plasma PL compared to healthy subjects. PL levels seem to decline during disease as patients with advanced cancer show even lower levels [4, 5]. In the present project the main focus is on the PL Lysophosphatidylcholine (LysoPC), which is a common plasma constituent with a concentration of approximately 300 μM in healthy persons [3, 6–8]. Blood plasma contains a mixture of LysoPC species carrying both saturated or unsaturated fatty acids (FA), with about 40 to 44 % unsaturated LysoPC species [9, 10].

Although a few studies, such as from Okita et al. [11] refer to increased LysoPC levels in patients suffering from cancer, the majority of studies focusing on LysoPC in cancer patient reported reduced LysoPC levels associated with malignant diseases. Colorectal cancer patients [10] as well as patients suffering from renal cell carcinoma [12] show significantly reduced LysoPC plasma levels. LysoPC concentrations were already decreased in the early stages of the disease of digestive tract tumours and renal cell carcinoma [8]. In a study with 59 patients suffering from various tumour entities (breast, prostate, lung, lymphoma, gastrointestinal), their reduced LysoPC levels have been found to be associated with increased parameters of inflammatory processes (CRP, albumin-reduction) as well as with severe weight losses [6]. *In vitro* studies confirmed that the tumour cells might be responsible for the increased LysoPC metabolism. It was reported that B16.F10 mouse melanoma cells *in vitro* rapidly remove exogenously added LysoPC from the supernatant [13]. The observed LysoPC removal appeared as an extremely fast, and for repeated exogenous administrations, unsaturable process. In these experiments, tumour cells were incubated with LysoPC carrying the saturated FA C17:0 (450 μM). Concordant with the decrease of LysoPC in cell culture supernatant, a strong increase of the LysoPC bound saturated FA (C17:0) was observed in cellular lipids from about 5 % to more than 50 % within 72 h of incubation [13]. Furthermore, this induced functional consequences, since an *ex vivo* pre-incubation of B16.F10 cells with saturated LysoPC led to a reduction by about 50 % in lung metastatic spread compared to untreated B16.F10 cells [13]. It was postulated that the strong increase of saturated FA and the subsequent decrease of ω-6 polyunsaturated fatty acids (PUFA) in the cellular lipids caused by the saturated LysoPC species impede the generation of lipid second messengers which are required for metastatic processes [14, 15]. Mechanistic consequences of tumour cell treatment with saturated LysoPC species were attenuated tumour cell adhesion and motility, shown under *in vitro* conditions. Pronounced morphological and functional surface changes were detected in cells treated with saturated LysoPC, which might contribute to the anti-metastatic effect by preventing integrin and selectin binding functions, but not affecting the expression levels of these adhesion receptors [13].

However, the molecular mechanisms of anti-metastatic activity were not understood and it remains open whether this is a peculiarity of the saturated nature of the LysoPC used in this study. Consequently it is questionable whether those effects can be transferred to the physiological LysoPC situation considering that more than a third of physiological LysoPC species carry unsaturated FA. To provide an insight into the underlying mechanisms of this area of LysoPC metabolism by tumours and potential consequences for metastatic spread, this study aims to address three main questions:

- Is the massive uptake and metabolism of LysoPC, as previously shown, a feature of melanoma cells, or a general characteristic of solid tumour cells and tumours of haematogenous origin?
- What is the fate of the LysoPC molecules in tumour cells, and is there a dependency on the saturation of the LysoPC bound FA, focusing on saturated and mono-unsaturated LysoPC species?
- If LysoPC indeed can affect the metastatic spread, can LysoPC levels be modified *in vivo* to use LysoPC or LysoPC precursors as active agents to interfere with metastatic properties of tumours?

## Results

### LysoPC removal by solid tumour cells and FA incorporation into cellular lipid pools

**Fig. 1:**
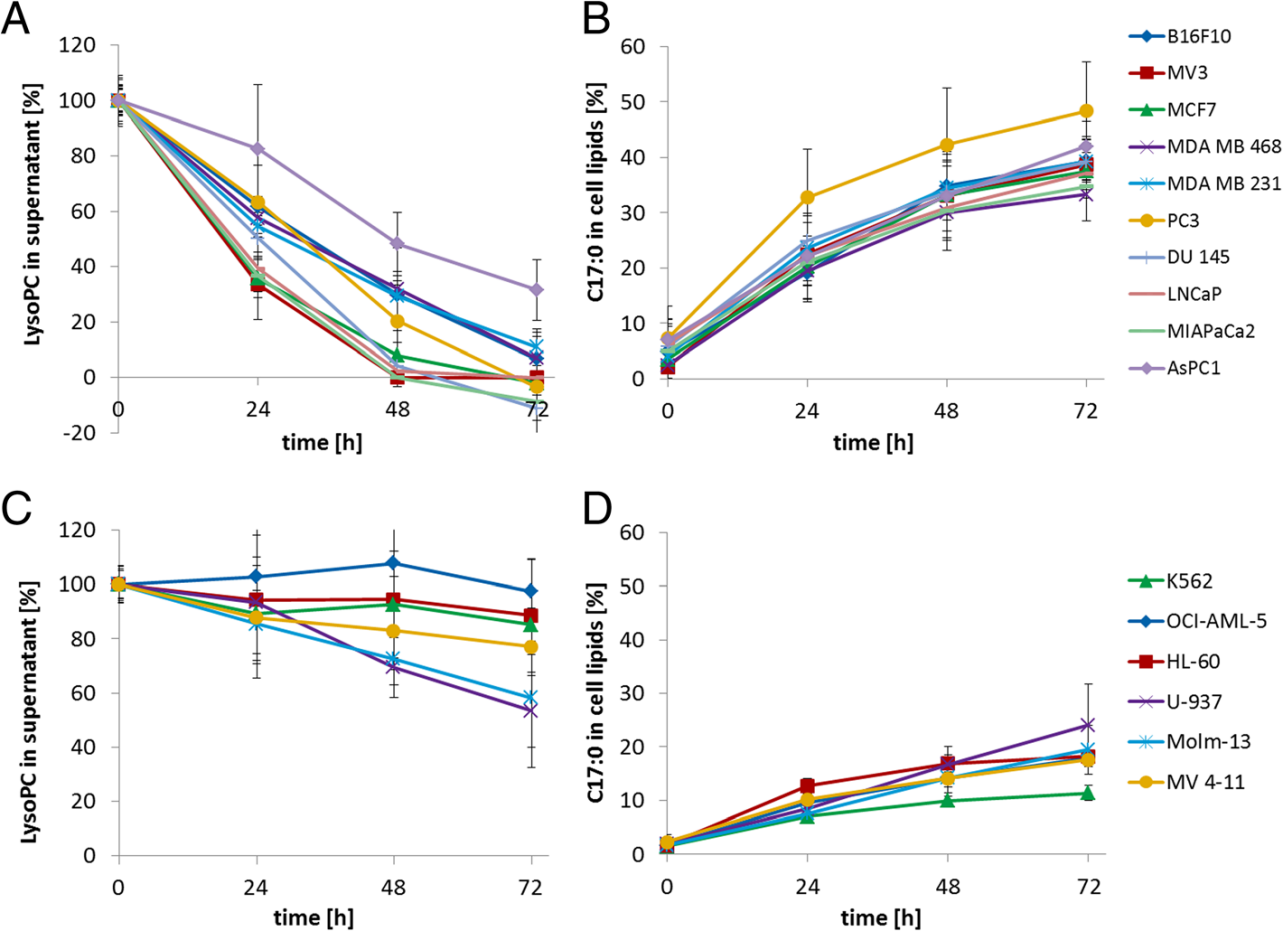
LysoPC removal and FA incorporation by solid tumour cells and leukaemic cells. **a**: Removal of exogenously added LysoPC 17:0 (450 μM) in cell culture supernatants of solid tumour cells (*n* = 3 measurements). **b**: Time course of cellular FA C17:0 ratio of LysoPC 17:0 treated solid tumour cells (450 μM). **c**: Time course of the degradation of LysoPC 17:0 (450 μM) from cell culture supernatant of leukaemic cells (*n* = 6 measurements). **d**: Incorporation of FA C17:0 into cellular lipids due to LysoPC treatment of leukaemic cells (*n* = 3 measurements). Changes in FA C17:0 contents are shown in % of total FA in time course experiments

In the LysoPC- or FFA-supplemented media, the proliferation rates of all tested tumour cell lines were statistically identical to the proliferation in non-supplemented BSA (control) medium (BrdU-assay, data not shown) proving no cytotoxic or growth reducing effects of LysoPC in the following assays. In media containing LysoPC (C17:0) and BSA, LysoPC was rapidly eliminated by all ten tested solid tumour cell lines (Fig. 1a). However, some cell lines (MV3, MIA PaCa2, Du145, and LNCaP) eliminated the total amount of LysoPC very fast (complete elimination within 48 h), while the LysoPC-elimination by other cell lines (MDA MB 231, MDA MB 468, PC3, B16.F10, and MCF7) was somewhat slower (complete elimination after 72 h). AsPC1 cells showed the slowest LysoPC-elimination.

Simultaneous with the rapid degradation of LysoPC, a strong increase of the LysoPC bound FA C17:0 could be observed in the cellular lipids. While the physiological content of FA C17:0 in the cellular lipids is about 5 % of total FA, incubation with LysoPC 17:0 caused an increase by about 30 to 50 % of total FA (Fig. 1b). The strongest rise was found in PC3 cells; AsPC1 cells showed the smallest rise which was about 30 % after 72 h of incubation. According to the increasing amount of C17:0 in cellular lipids, the relative amount of the other analysed FA decreased.

### LysoPC removal and FA incorporation into cellular lipid pools of leukaemic cells

Compared to the solid tumour cell lines, LysoPC-removal in the supernatant of leukaemic cell lines was much slower (Fig. 1c). In accordance to that, the incorporation of the administered C17:0 is less pronounced. After 72 h, only an average ratio of 18 % C17:0 was reached in the cellular lipids of the leukaemic cells while the average ratio in the solid tumour cells was twice as high (41 %). It has to be mentioned that the differences between leukaemic and solid tumour cells may be even more pronounced as shown here, since due to experimental reasons, higher cell counts were used in the experiments with leukaemic cells as in the experiments with solid tumour cells.

### Metabolism of saturated and unsaturated LysoPC species by solid tumour cells

**Fig. 2:**
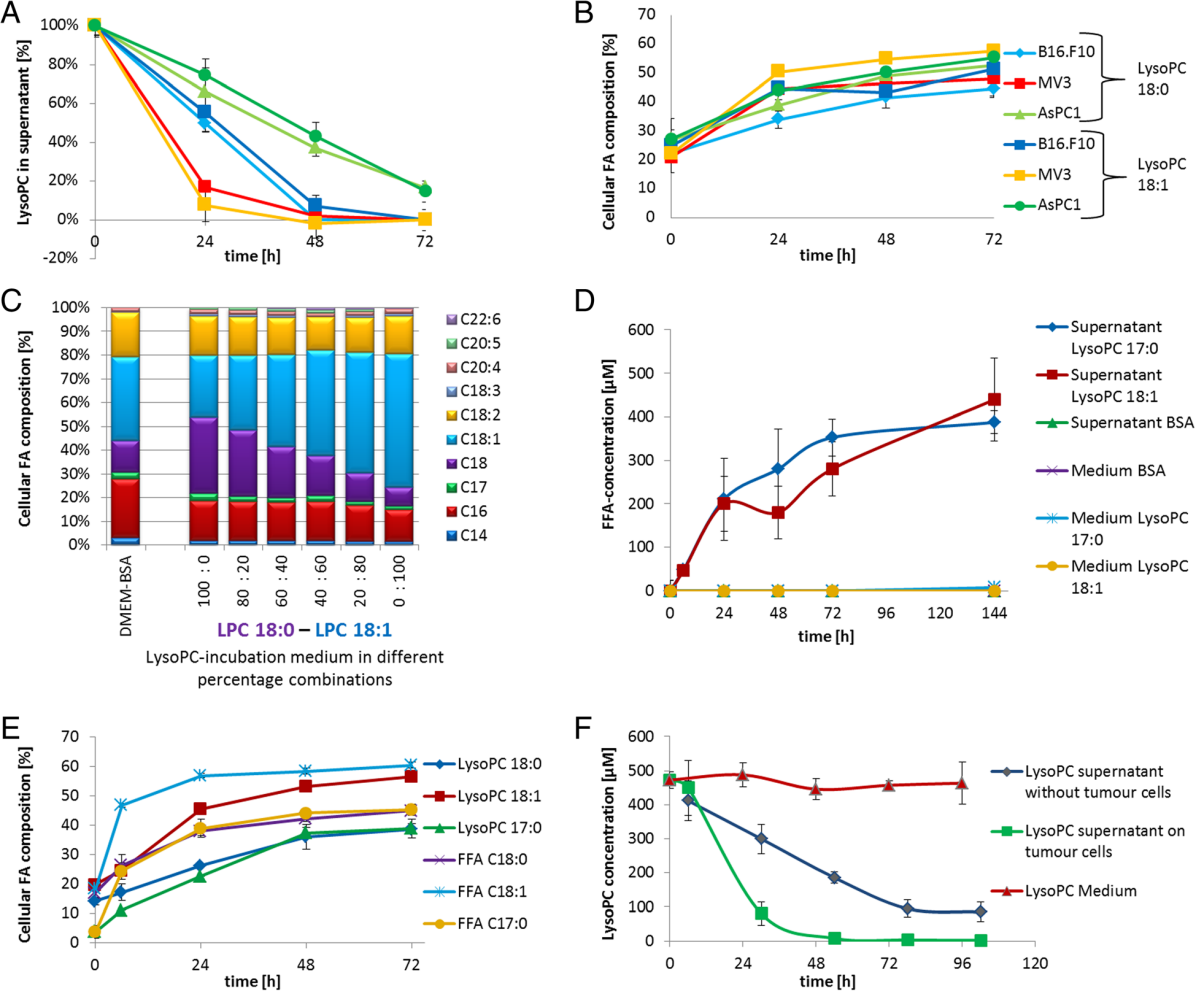
Metabolism of saturated and unsaturated LysoPC and FFA species by solid tumour cell. **a**: Time course removal of exogenously added saturated and unsaturated LysoPC species (18:0 and 18:1) in cell culture supernatants of solid tumour cell lines B16.F10, MV3, and AsPC1 cells. **b**: Changes of cellular lipid FA ratios of FA C18:0 and FA C18:1 (in % of total FA) in LysoPC (C18:0 and C18:1) treated cells. **c**: FA composition in B16.F10 cells after treatment with two LysoPC species simultaneously in different ratios (for 48 h). **d**: Release of FFA to cell culture supernatants, analysed by an enzymatic FFA assay. B16.F10 mouse melanoma cells were incubated with LysoPC 17:0, C18:1, and BSA medium. Media without contact to cells were analysed as control. **e**: Comparison of incorporation of different LysoPC species and the corresponding FFA into B16.F10 mouse melanoma cells. Increases of the respective FA are shown in time course experiments. **f**: Comparison of LysoPC degradation in B16.F10 tumour cells (green) with the degradation in supernatant after removal of the tumour cells (blue), and LysoPC containing medium without cell contact (red). B16.F10 cells were cultivated in LysoPC 17:0 medium. After 6 h, supernatant was separated from the cells and further incubated (blue)

**Fig. 3:**
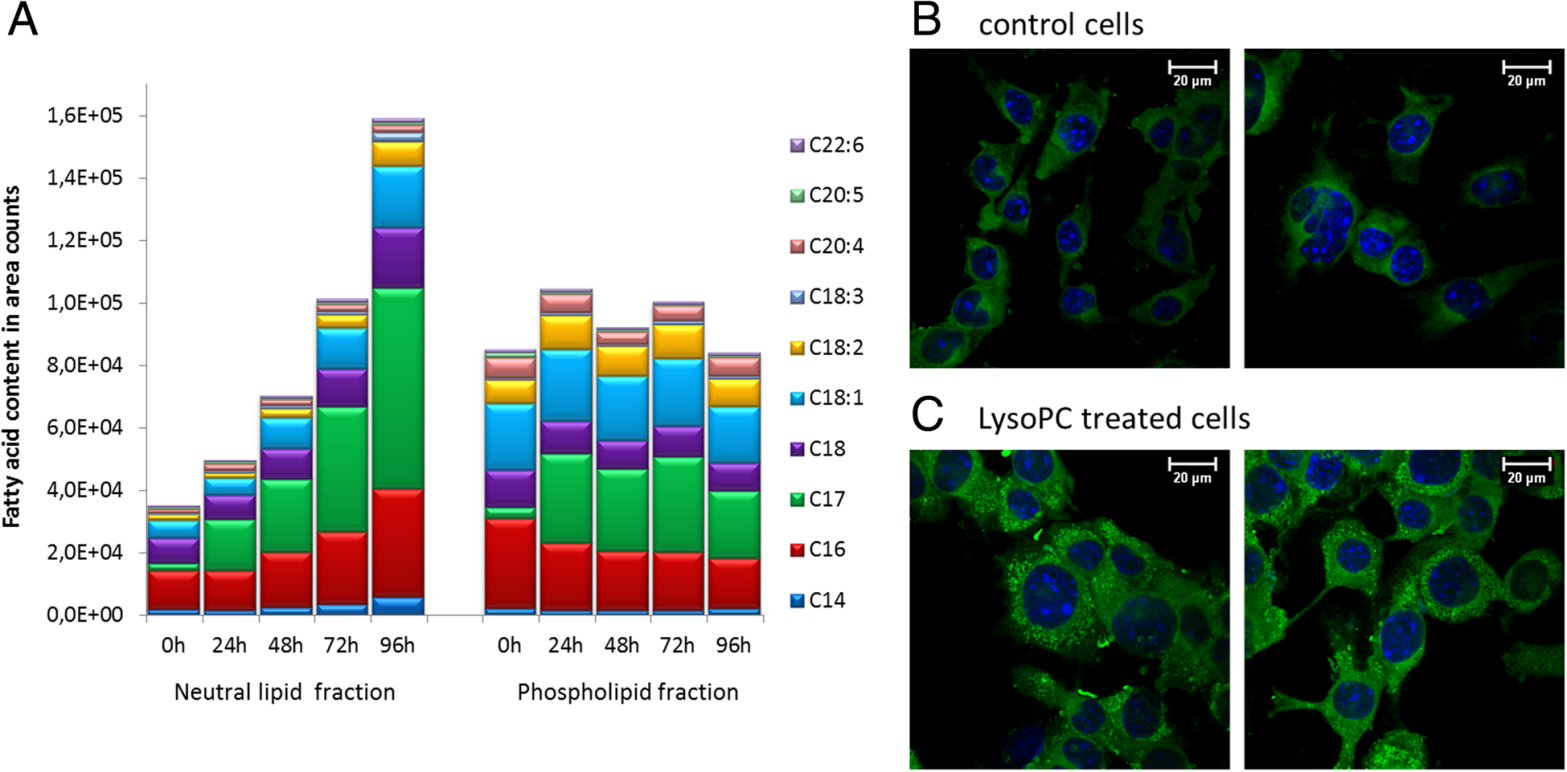
FA incorporation into PL and neutral lipid fraction and LD formation. Changes in the FA distribution of B16.F10 tumour cells treated with LysoPC 17:0 over 5 days. **a**: Lipid fractions, neutral lipids and PL were individually analysed and total FA are displayed as area counts of gas chromatography measurement (*n* = 4 measurements). **b**/**c**: Fluorescence Laser Scanning Microscope images of B16.F10 melanoma cells after staining with DAPI (blue) and BODIPY (green). **b**: Cells cultivated in control medium DMEM-BSA, **c**: Cells treated with LysoPC 17:0 (450 μM) for 16 days

LysoPC with saturated and unsaturated FA (C18:0 and C18:1) were identically degraded by three selected solid tumour cell lines. Carrying a saturated FA seems not to play a decisive role in LysoPC degradation (Fig. 2a). Comparison of the FA incorporation kinetics in the cellular lipids also revealed no differences between the saturated and unsaturated LysoPC species (Fig. 2b). In addition to that, simultaneous application of LysoPC 18:0 and 18:1 in various ratios showed that neither the saturated nor the unsaturated FA was preferentially taken up by the tested cell line (Fig. 3c).

Using B16.F10 cells as a representative cell line, it could be shown that simultaneous with the degradation of saturated or unsaturated LysoPC in the supernatant, the respective LysoPC bound FA was released into the supernatant (Fig. 2d). Incubation without LysoPC (only BSA-medium), or incubation of LysoPC containing media without cells, did not result in a FA increase, suggesting that lysophospholipase (LysoPLA)-activity is associated with the cells.

Further investigations showed that the degradation of LysoPC continued even without cell contact. Therefore LysoPC medium was pre-incubated on cells for 6 h and further incubated after separation of the cells. The degradation rate in the cell free supernatant was about 40 % of the degradation rate in the presence of the cells, indicating that the LysoPLA-activity of the tumour cells is partly released into the cell culture supernatant (Fig. 2f). Regarding LysoPLA, it was shown that the products of LysoPC cleavage, FFA and GlyceroPC, had no inhibitory effect on the LysoPLA-activity (no product inhibition), even when applied in very high concentrations (450 μM).

To explore whether LysoPC cleavage to FFA outside the cells contributed to the observed LysoPC-induced changes, we compared the modification of the cellular lipid composition, induced by saturated or unsaturated LysoPC species and the corresponding FFA, respectively (Fig. 2e). Indeed, FFA induced a very similar but somewhat faster change of the cellular FA composition compared to the corresponding LysoPC species; the ratios of the applied FA reached the same level after 72 h. This finding strengthen the assumption that FA uptake is dependent on LysoPC cleavage also explains the somewhat slower cellular FA incorporation caused by LysoPC.

### FA incorporation into PL and neutral lipid fraction

As shown in Fig. 3a, incubation of B16.F10 cells with LysoPC 17:0 changed both the lipid composition of the membrane PL and of the neutral lipids. While the amount of the PL-fraction remained rather constant during LysoPC incubation, the amount of the neutral lipid fraction increased fourfold.

The increase of the neutral lipids in B16.F10 cells is accompanied by an impressing increase of intracellular lipid droplets (LD), as visualised by fluorescence confocal scanning laser microscopy (Fig. 3c). In control cells, only a green shimmer but no differentiated spots could be recognised (Fig. 3b).

### Effects of saturated and unsaturated LysoPC species on membrane fluidity and cell migration

**Fig. 4:**
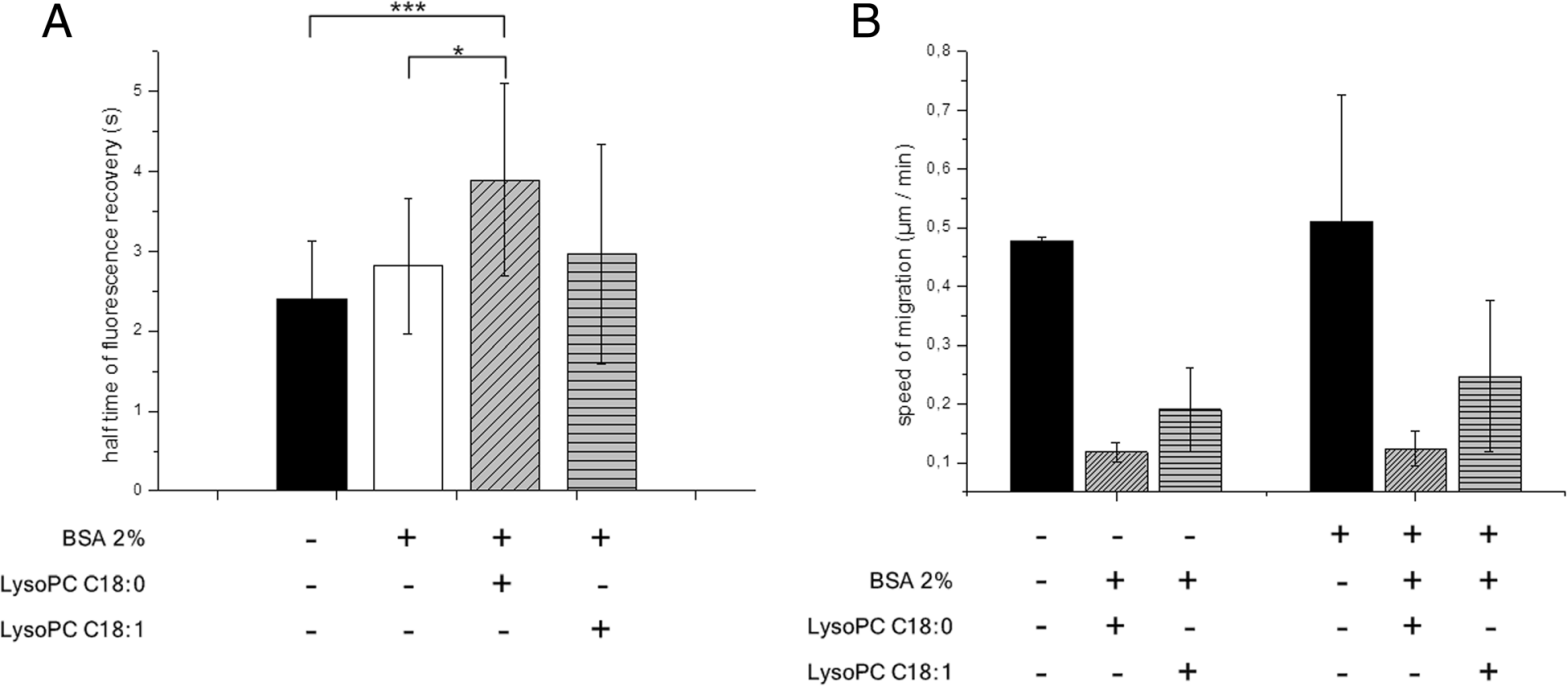
Effects of saturated and unsaturated LysoPC species on membrane fluidity and cell migration. **a**: Measurement of half-life of fluorescence recovery of B16.F10 cells with NBD-PE C18:1 as the fluorescent probe at a temperature of 37 °C. Higher values indicate slower lateral movement and lower membrane fluidity. Control cells in DMEM (*n* = 17), BSA cells, LysoPC 18:0, and LysoPC 18:1 (*n* = 17-18), mean ± SD; * *p* ≤ 0.05, *** *p* ≤ 0.001. **b**: Speed of two dimensional cell migration detected by scratching assay onto an uncoated or collagen-coated surface of B16.F10 cells untreated or pre-incubated with BSA containing LysoPC 18:0 or LysoPC 18:1 media (450 μmol/l); (*n* = 2)

Compared with control cells, treatment of B16.F10 cells with saturated LysoPC 18:0 showed reduced membrane fluidity indicated by lateral lipid membrane mobility measured by the fluorescence recovery after photobleaching (FRAP) technique. Treatment with unsaturated LysoPC 18:1 had only a minor effect resulting in a slightly, but not significantly reduced membrane fluidity (Fig. 4a). This has functional consequences for cell migration as indicated in Fig. 4b showing data of scratching assays. LysoPC 18:0 pre-incubated cells display the slowest migratory capacity on both, uncoated or collagen-coated surfaces compared to untreated or LysoPC 18:1 pre-treated cells. Despite of the minor effect of LysoPC 18:1 on membrane fluidity (Fig. 4a), the mono-unsaturated LysoPC also induced a statistically significant attenuated migration on both surfaces, although less pronounced compared to LysoPC 18:0.

### *In vivo* effects of pre-incubated B16.F10 cells with saturated and unsaturated LysoPC and FFA species

**Fig. 5:**
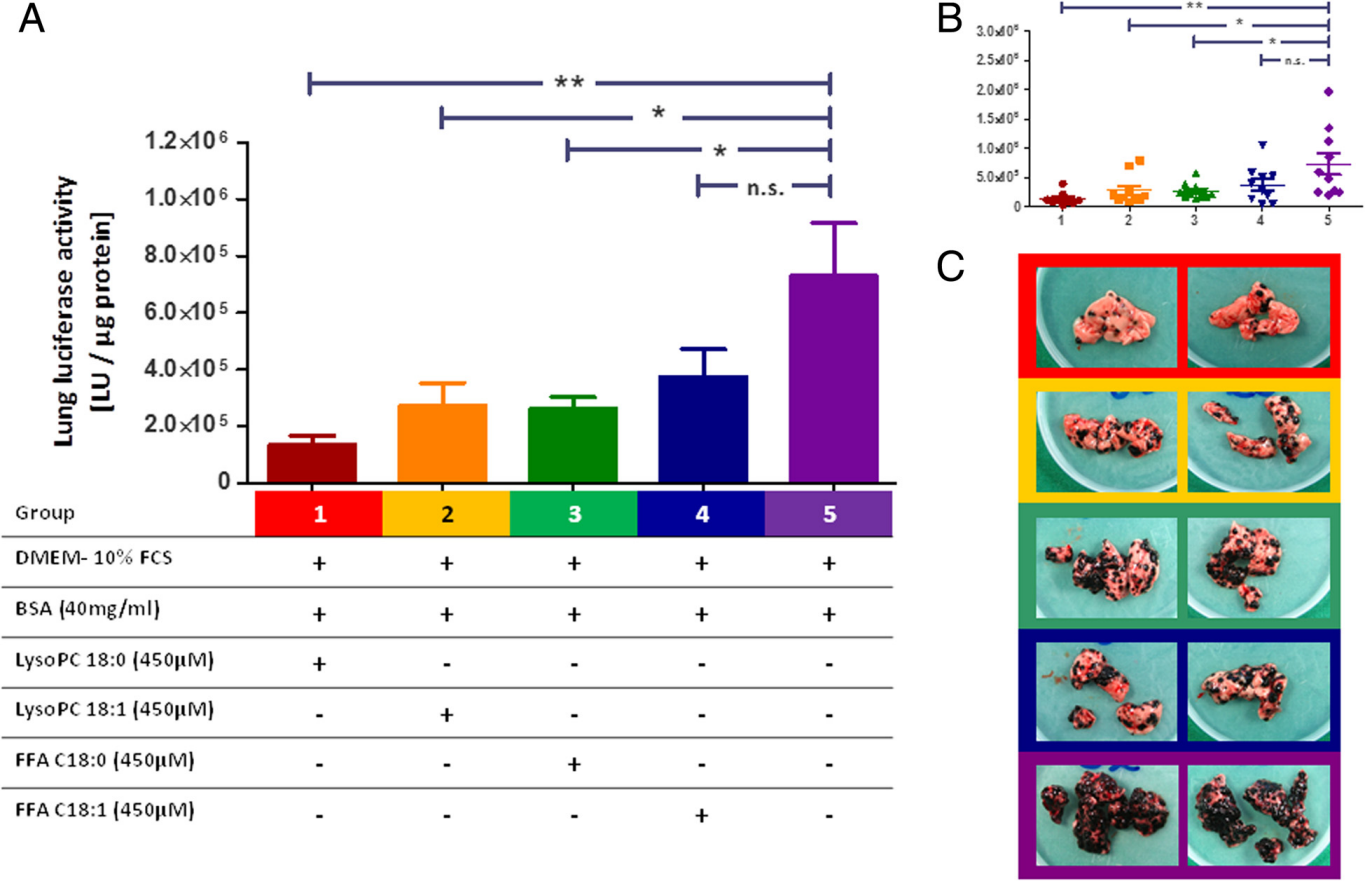
In vivo effects of pre-incubated B16.F10 cells with saturated and unsaturated LysoPC and FFA species. Luciferase activity of homogenised mouse lungs analysed by luciferase assay (day 18). **a**: mean values ± SEM of mice which received different pre-treated B16.F10 cells (n = 10 for each group). **b**: single values of each mouse ± SEM. Values compared by ANOVA: n.s. *p* ≥ 0.05, * *p* ≤ 0.05, ** *p* ≤ 0.01. **c**: Pictures of mouse’s lungs after necroscopy, examples of two mice per group are shown

All mice tolerated the injections of the tumour cells pre-treated for 10 days with medium supplemented with LysoPC 18:0, LysoPC 18:1, FFA C18:0, or FFA C18:1, as well as the BSA treated control cells, very well. Mice began to lose weight 15 days after tumour cell injection, thereby no significant differences between the various groups were found. Macroscopic visual lung metastasis after 18 days (Fig. 5c) revealed that the mice receiving control cells, incubated only with BSA, had the highest metastatic activity. This was confirmed by measuring the luciferase activity of the homogenised lungs. The lowest values, corresponding to the lowest metastatic burden were found in the lungs of mice carrying cells incubated with LysoPC 18:0 cells (80 % reduction). Pre-incubation with LysoPC 18:1 and FFA C18:0 also caused significant reduction of metastasis like lung invasion (reduction of 60 % and 65 %, respectively), while FFA C18:1 had no significant effect (Fig. 5a and b).

### LysoPC plasma level in healthy mice and tumour bearing mice

**Fig. 6:**
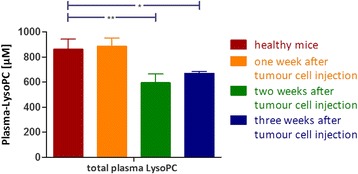
LysoPC plasma level in healthy mice and tumour bearing mice. Total LysoPC plasma levels of healthy mice (*n* = 4) and mice intravenously injected with B16.F10 melanoma cells; one (*n* = 3), two (*n* = 3) and three (*n* = 3) weeks after injection of tumour cells. LysoPC levels were determined by HPLC MS/MS analysis. Values compared by ANOVA: * *p* ≤ 0.05, ** *p* ≤ 0.01

Compared to healthy mice, LysoPC plasma levels in mice were significantly reduced two or three weeks after injection of B16.F10 melanoma cells while this difference was not yet detectable already one week after injection of the tumour cells. No differences were found between healthy mice and tumour bearing mice one week after tumour cell injection (Fig. 6). With regard to the different LysoPC species, the most pronounced decrease was found for LysoPC 16:0. There was also an apparent decrease in LysoPC 18:2 and LysoPC 20:4; however these differences were not significant.

### Manipulation of the LysoPC plasma levels and its effects on metastatic spreading

With the aim to reduce metastatic spreading by increasing the LysoPC plasma level and/or by changing the ratio of the different LysoPC species towards saturated LysoPC, different approaches were investigated: chow supplemented with saturated PC containing mostly C18:0, s.c. injection of saturated LysoPC, and injection of liposomes containing the same saturated PC.

Chow supplemented with saturated PC did not cause any significant changes compared to non-supplemented chow.

Concerning s.c. injection of LysoPC 17:0, 30 min after application, a minimal increase of this LysoPC species could be observed (10.2 ± 1.8 μM; *n* = 5) compared to control mice (6.1 ± 2.8 μM; *n* = 4). However, the total LysoPC levels did not show any increase compared to control mice (765.0 ± 129.2 μM vs. 740.2 ± 45.7 μM, respectively).

**Fig. 7:**
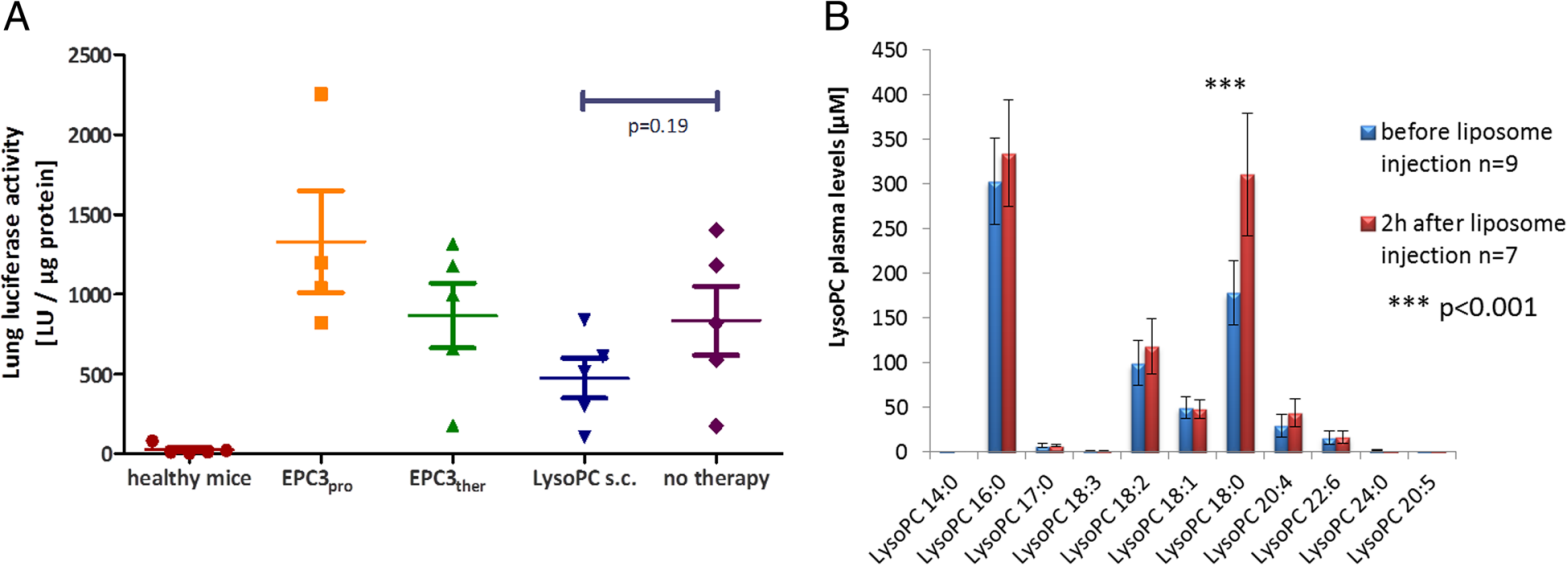
Manipulation of the LysoPC plasma levels and its effects on metastatic spreading: **a**: Luciferase activity of mouse lung homogenates from mice without tumour cell injection (healthy control mice, *n* = 5) and mice injected with B16.F10 tumour cells receiving different treatments: mice receiving supplemented chow starting one month before tumour cell injection (EPC3pro, *n* = 4) and starting after tumour cell injection (EPC3ther, *n* = 5); mice with LysoPC s.c. injections (*n* = 5) and tumour control mice (no therapy, *n* = 5). **b**: Mean values ± SD of plasma LysoPC levels before (*n* = 9) and after (*n* = 7) i.v. injection of liposomes, plasma LysoPC levels determined by HPLC MS/MS analysis

Investigating the LysoPC species and levels 2 h after injection of high doses of liposomes consisting predominantly of saturated PC showed a significant increase (p < 0.001) of LysoPC 18:0 (179 ± 36 μM before vs. 311 ± 69 μM after injection, *n* = 7). The other analysed LysoPC species were not significantly changed (Fig. 7b).

Next, the impact of these treatment regimens on metastases was investigated. Consistent with the finding that PC-supplemented chow caused no change of plasma LysoPC levels or composition, PC-supplemented chow given before and after tumour cell injection (EPC_pro_) or given only after tumour cell injection (EPC_ther_) had no effect on metastatic spreading three weeks after tumour cell injection (Fig. 7a, row 2 & 3).

S.c. injections of LysoPC 17:0 in addition to the injection of the B16.F10 cells (six times in 12 h intervals, starting 23 h before injection of tumour cells) caused a slight but not significant decrease of metastatic spreading compared to animals receiving no treatment (Fig. 7a, row 4 & 5). The anti-metastatic effects of liposome injections were not investigated here, as the anti-metastatic effects have already been shown [16, 17].

## Discussion

Previous studies have shown that B16.F10 melanoma cells *in vitro* rapidly remove exogenously added saturated LysoPC from the supernatants and that the LysoPC bound saturated FA are incorporated within the cellular lipids. Both correlate with reduced adhesion properties of the cells *in vitro* and strongly reduced metastatic spreading of the cells *in vivo* [13].

Here we provide a first insight into the underlying mechanisms and the potential interplay between LysoPC and tumour metastasis. We demonstrate that the rapid removal of LysoPC is not only a peculiarity of melanoma cells but also seems to be a general characteristic of solid tumour cells. We propose a model of extracellular LysoPC degradation and cellular FFA uptake that appears independent of the saturation of LysoPC. Based on our findings the potential anti-metastatic activity of saturated as well as mono-unsaturated LysoPC appears as an attractive pathway for a therapeutic interference. These aspects will be discussed below.

### LysoPC removal by different tumour cell lines

**Fig. 8:**
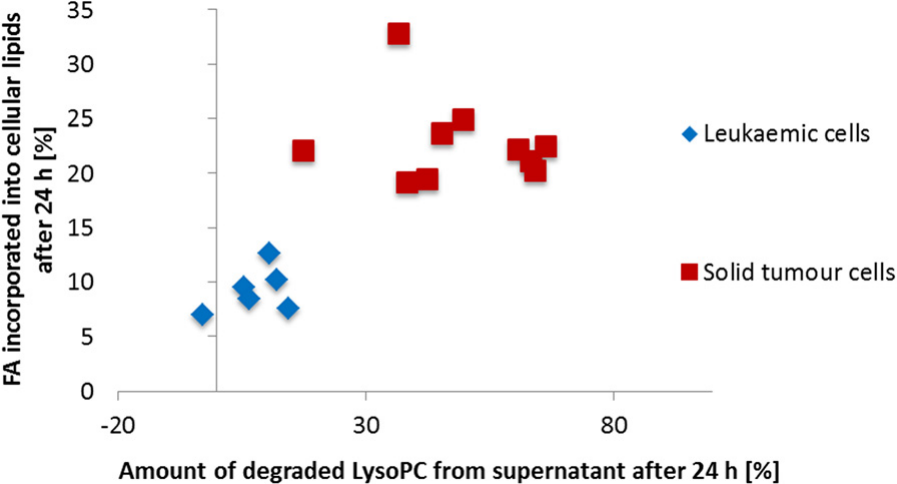
LysoPC removal and FA incorporation by different tumour cell lines. Correlation of LysoPC elimination from cell culture supernatant and incorporation of the respective FA into cellular lipids after 24 h of incubation. Blue: leukaemic cell lines, red: solid tumour cell lines, (including human melanoma, breast cancer, prostate cancer, and pancreatic cancer cells)

Strongly reduced LysoPC plasma levels can be observed in patients suffering from various tumour entities [6, 8, 10]. Investigating ten different epithelial tumour cell lines and six leukaemic cell lines, a very rapid decrease of LysoPC from cell culture supernatant and the incorporation of the LysoPC bound FA into the cellular lipid pools was exclusively observed for the epithelial tumour cells (Fig. 8). Interestingly, a study comparing the LysoPC degradation in HUVEC, monocytes, erythrocytes and platelets, found that HUVEC effectively degrade extracellular LysoPC to FFA, while monocytes clearly showed less LysoPC degrading activity. Erythrocytes and platelets had nearly no LysoPC degrading activity [18]. Thus, the rapid removal of extracellular LysoPC by solid tumour cells with metastatic potential might be a general characteristic of these cells and can be discussed as a necessary but surely not sufficient feature for metastatic spreading.

The ability of the tested epithelial tumour cells to rapidly cleave extracellular LysoPC might also explain the reduction of the LysoPC plasma levels in most patients with epithelial tumours as well as the negative correlation of the LysoPC levels with the progress of the disease [6]. Concordant to the decrease of LysoPC in patients we found a slight but significant reduction of LysoPC in mice with B16.F10 lung metastases. The lower reduction in mice compared to humans with cancer can be explained by the twofold higher plasma LysoPC levels in mice (600–700 μM) compared to humans, as shown in this and other studies [19, 20], indicating a higher LysoPC turnover in mice which might better compensate the tumour induced LysoPC decreases.

### LysoPC metabolism of tumour cells

**Fig. 9:**
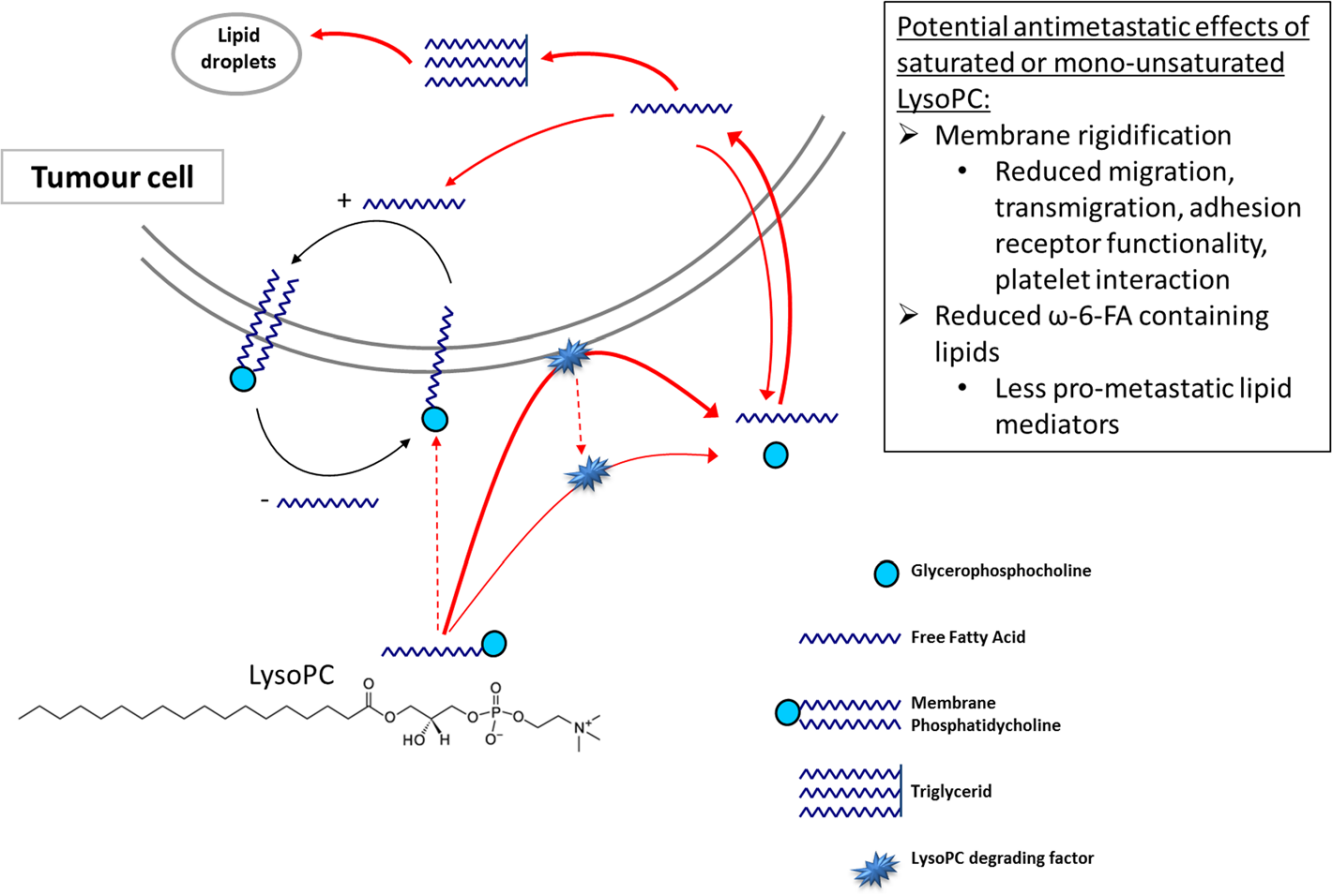
Proposed uptake/metabolism of LysoPC in tumour cells. The majority of LysoPC is extracellularly degraded to GlyceroPC and FFA by a LysoPC degrading factor. This factor – probably a LysoPLA – is partly released into the supernatant of the tumour cells. The resulting extracellular FFA can subsequently be taken up and incorporated into membrane PL and neutral lipids. Excess of neutral lipids can be stored as LD. Another possible uptake route for LysoPC is its incorporation into the cellular membrane as a whole molecule, where it becomes part of the Lands’ Cycle: the deacylation and reacylation process

Our studies concerning the fate of the extracellular LysoPC and the increase of the LysoPC bound FA in the cellular lipids revealed (i) that incubation with saturated LysoPC species as well as the corresponding FFA results in an almost identical incorporation of the respective FA within the cellular lipids, and (ii) that removal of LysoPC from the cellular supernatant is accompanied by a corresponding increase of extracellular FFA. Obviously, the main mechanism for the cellular uptake of LysoPC bound FA consists of a rapid cleavage of the sn-1-ester bond of LysoPC by a LysoPLA activity followed by the rapid cellular uptake of the resulting FFA, supported by the high FFA gradient between the intracellular and extracellular environment and the rapid transmembrane movement of FFA [21]. High LysoPLA activities have been observed in certain mammalian cells and tissues (e.g. liver, gastric mucosa, kidney, brain, lung, and macrophages) [22, 23]. Data for LysoPLA activities in tumour tissues/cells are not yet available. The proposed LysoPLA activity that we found in tumour cells is at least partly released into the cell culture supernatant, as LysoPC degradation continues when the supernatant is further incubated cell-free. A model summarizing these processes is suggested in Fig. 9.

The most obvious way for LysoPC molecules to enter the cell so far known is a passive uptake into the cell membrane, involving a flip to the inner membrane layer, where it may become part of the Lands’ cycle [24]. This is in contrast to the path suggested here, to take up the LysoPC-derived FFA but not the LysoPC molecule itself.

Within the Lands’ cycle, LysoPC is reacylated to PC and PC is deacylated to LysoPC, thus introducing the LysoPC bound FA into the membrane PLs. However, an additional direct uptake of LysoPC cannot be excluded from our data, at least at the beginning of the incubation when extracellular LysoPC is not yet degraded and its amount greatly exceeds the total lipid amount of the cells.

### Increase of neutral lipids and formation of LD

Excess extracellular FFA, established either by the rapid extracellular cleavage of LysoPC or simply by adding FFA, results in the rapid formation of neutral lipids and LD. LD have a surface consisting mainly of PC and are the major cellular organelles responsible for the storage of neutral lipids [25, 26]. Neutral lipids and subsequently FFA can be mobilised for energy generation via ß-oxidation [27]. Furthermore, LD are a repository for membrane building blocks, including PL and sterols [26].

Bozza et al. [28] reported the finding of an increased number of LD in cancer tissues. As LD play a role in inflammation and neoplastic processes, they are highly regulated organelles. Various enzymes involved in eicosanoid synthesis can be found localised at LD. Thus, LD are sites of eicosanoid generation and particularly active in the metabolism of arachidonyl lipids [28]. Significant correlations have been found between LD and enhanced formation of COX-derived eicosanoids [29, 30] thereby possibly contributing to pro-metastatic activity. However, in contrast to physiological LysoPC carrying a mix of various FA including pro-metastatic ω-6 PUFA (C18:2 and C20:4), in our study the formation of LD is predominantly caused by saturated or mono-unsaturated LysoPC, which might contribute to the observed anti-metastatic effects by the reduction of pro-metastatic ω-6 PUFA in LD [14, 15]. The probable corresponding pro-metastatic effects of LysoPC-species carrying ω-6 PUFA was not the issue of this study and has to be investigated in future studies.

### Different saturation grades of LysoPC and FFA species and their impact on membrane fluidity

So far, the anti-metastatic effect of LysoPC was observed using saturated LysoPC species [13]. Here we found that saturated and at least mono-unsaturated (C18:1) LysoPC were identically eliminated from the supernatant of three representative cell lines and comparably increased the FA-content in the cellular lipid pools. The use of various mixtures of LysoPC 18:0 and 18:1 for the cell cultivation did not reveal a preferred metabolism for either LysoPC species.

The increase of FA 18:0 within the cell membranes was accompanied by reduced membrane fluidity. Membrane fluidity determines various biological processes such as adhesion, receptor activity and cell motility [31, 32] and thus appears as functional link to explain attenuated metastatic spread, as shown previously [13] (Fig. 9). Interestingly, tumour cell incubation with the unsaturated LysoPC 18:1 and the subsequent increase of oleic acid in the cellular PL also resulted in a decrease of the membrane fluidity, however, not as distinct as for incubation with the saturated LysoPC 18:0. This can be explained by the fact that oleic acid carrying only one double bond has not the same effect on membrane fluidity as higher unsaturated FA. This explanation of the effects of LysoPC 18:0 and 18:1 on membrane fluidity is further supported by our findings that both LysoPC 18:0 and 18:1 had a clear effect on cell migration, and again, the effect was somewhat weaker for LysoPC 18:1.

### Effects of saturated and unsaturated LysoPC/FFA on metastatic spread *in vivo*

Investigating the metastatic behaviour of LysoPC and FFA pre-treated tumour cells in an *in vivo* mouse model revealed the strongest reducing effect on metastatic spread for the saturated LysoPC 18:0, the effects of the mono-unsaturated LysoPC 18:1 were still impressive but less pronounced. LysoPC and its corresponding FFA, respectively, showed comparable effects on metastatic spread, but the effects of the FFA were somewhat weaker (effect of FFA 18:1 missed significance), implicating that the anti-metastatic effect was at least partly caused by the entire LysoPC molecule, but the underlying mechanism remained unclear. It should be mentioned that there are no significant differences between the treatment groups.

In agreement with our results, an anti-tumoural effect of oleic acid (C18:1) was seen in epidemiological and in animal studies [33]. The effects were explained by inhibition of proliferation in different tumour cell lines [34] or suppression of oncogenes which play a role in invasive progression and metastasis [35]. Reduced synthesis of arachidonic acid derived eicosanoids were also considered to inhibit the growth of tumours [36]. In human studies the “Mediterranean diet”, rich in oleic acid displays protective effects against cancer [37]. Furthermore, the saturated stearic acid (C18:0) has been described to have anti-cancer properties *in vitro* and *in vivo*, targeting tumour proliferation, migration and tumour invasion. Mice with orthotopically growing breast cancer showed reduced tumour size (approximately 50 % reduction) and partially reduced lung metastases when they were supplied with a diet rich in saturated FA [38].

### Prospects for affecting LysoPC levels in term of anti-metastatic approaches

Following the idea that the increase of plasma LysoPC might influence metastatic cells also *in vivo*, we investigated and compared different ways to increase the plasma levels of saturated LysoPC and its effects on metastatic tumour growth in mice. Since LysoPC cannot directly be injected intravenously into mice due to its haemolytic activity [39], three alternative approaches were tested: the oral application of the LysoPC precursor PC, s.c. LysoPC injection and i.v. injection of liposomes consisting of saturated PC.

Dietary PC (2 % in the chow) was chosen since PC can be degraded to LysoPC intestinally and subsequently can be absorbed [20]. However, PC enriched feed had no effects on LysoPC plasma levels of mice and no effect on metastatic growth, neither if mice received the feed as pre-treatment nor as therapeutic treatment. The lacking effects might especially be due to a rapid hydrolysis of LysoPC to FFA in the intestine prior uptake [40].

The s.c. injection of LysoPC (2 mg/kg bodyweight) only results in a slight increase of LysoPC plasma levels. This was in contrast to the observations of Yan et al. [19] who reported a LysoPC increase by 11 % one hour after injection (577 ± 17 μM to 633 ± 17 μM). Nevertheless, s.c. injection of LysoPC seems to reduce the metastatic spreading of tumour cells when mice were treated with saturated LysoPC in 12 h intervals, however, the effect was not significant. However, despite the fact that the differences were not significant, the observed reduction in metastatic spreading warrants future studies focusing on antimetastatic effects of LysoPC (s.c.), including different time schemes and more animals.

I.v. injection of liposomes containing saturated PC was expected to increase LysoPC plasma levels by hydrolysis of liposomal PC in the systemic circulation by enzymes physiologically metabolising lipoproteins (endothelial lipase, LCAT) [41, 42], or after accumulation in the tumour tissue by phospholipase A_2_ secreted by the tumour cells [43, 44]. In fact, two hours after injection of high doses of drug-free (empty) liposomes consisting mainly of hydrogenated egg-phosphatidylcholine (307.2 mg PC/kg, mainly Di-C18:0-PC [16, 17]) there was a significant increase in LysoPC 18:0 of about 130 μM. Future studies have to investigate how long this increase of LysoPC will last. In a recent study the same liposomes showed an impressive anti-metastatic effect in a mouse model with orthotopically implanted pancreatic tumour cells (MIA PaCa2) [17]. This effect of “empty liposomes” could be reproduced using another metastases model in mice, the pancreatic tumour cell line AsPC1 with the same experimental setup [16]. The effects of saturated LysoPC on metastases of MIA PaCa2 tumours were more pronounced than the effects on AsPC1-induced metastases, which correlates with our finding that MIA PaCa2 cells degrade LysoPC and take up the saturated FA twice as fast as AsPC1 cells. Taken together, our results provide an explanation for the very impressive anti-metastatic effects of empty liposomes in our mice studies. Thus, liposomal drug delivery at least has the chance to effectively combine chemotherapy for treating the primary tumour and additional anti-metastatic effects.

However, to put these findings into the right perspective, it has to be mentioned that the lipid dose which caused the described anti-metastatic effects and the strong increase in saturated LysoPC *in vivo* was rather high. The lipid doses, which are currently in use for registered liposomal formulations in oncology, are about an order of magnitude lower than the doses used in our experiments: The dose of the liposomal-hydrogenated PC used as carrier control in mice studies with liposomal Gemcitabine (GemLip: 6 mg/kg (MTD)) and which showed the above described anti-metastatic effects, was 307.2 mg/kg. In contrast, in comparable mice experiments with Caelyx®/Doxil®, only 43.2 mg/kg hydrogenated soy PC were used (which correspondents to 9 mg/kg liposomal Doxorubicin (MTD)) [45].

For patients suffering from metastatic breast or ovarian cancer, the recommended Caelyx®/Doxil® dose is 50 mg per m^2^ every four weeks, which corresponds to a PC amount of 240 mg/m^2^. Thus, this relatively low amount of saturated PC applied as liposomal phospholipid to these patients is most probably the reason why no extra “anti-metastatic” effect of the liposomal carrier has been yet described.

## Conclusions and outlook

Metastasis is a life-threatening complication of cancer and unfortunately to date there are hardly any therapeutic options for treating metastasis. The observations we made here lead us to the hypothesis that the rapid extracellular hydrolysis of LysoPC by metastatic tumour cells and the subsequent cellular uptake of the resulting FFA seems to be a necessary prerequisite for metastatic potential of epithelial tumour cells, allowing the cells to rapidly satisfy their high demand on various FA, for energetic purposes, for maintaining a certain membrane fluidity and probably also for generating pro-metastatic lipid second messengers. As a consequence, disturbing or inhibiting this process might be a promising way to reduce metastases, which should be investigated in future studies. Further experiments regarding the LysoPLA activity are required; this includes the inhibition of its secretion as well as inhibition of its LysoPC cleaving activity. Inhibiting LysoPLA should elucidate whether this has the potential to reduce metastasis. A first and promising step for reduced metastasis is presented here which indicates that manipulating the lipid metabolism of metastatic cells by supplying saturated or mono-unsaturated LysoPC species greatly reduced their metastatic potential. It appears promising that this effect could also be achieved *in vivo* by slightly increasing the ratio of saturated LysoPC species in the blood by applying liposomes.

## Methods

### Cell culture experiments

Solid epithelial tumour cells (from ATCC or DSMZ): B16.F10 mouse melanoma cells (CRL-6475), MV3 human melanoma cells (kindly provided by R. Zeisig, EPO Berlin, Germany); MCF7 (HTB-22), MDA-MB 231 (HTB-26), and MDA-MB 468 (HTB-132; human mamma carcinoma cells); MIA PaCa2 (CRL-1420) and AsPC1 (CRL-1682; human pancreatic cells); LNCaP (CRL-1740), DU 145 (HTB-81) and PC3 (CRL-1435; human prostate cancer cells) were cultured at 37 °C and 10 % CO_2_ in DMEM medium (Gibco Invitrogen, Germany) supplemented with 10 % fetal calf serum (FCS, Bio Whittaker, Lonza, Belgium). Leukaemia cell lines K-562 (CCL-243), OCI-AML 5 (DSMZ #ACC247), HL-60 (CCL-240), U937 (DRL-1593.2), Molm13 (DSMZ #ACC554), and MV4-11 (CRL-9591); growing as suspension cell cultures, were incubated at 37 °C and 5 % CO_2_ in RPMI 1640 medium with 10 % FCS.

For the LysoPC and FFA containing media, bovine serum albumin (BSA, PAA Pasching, Austria) were added to DMEM (10 % FCS medium) at a concentration of 40 mg/ml to prevent cytotoxic effects, mainly cell lysis, due to high concentrations of unbound LysoPC or FFA. Both, FCS and BSA, physiologically contained no additional LysoPC; this was verified by HPLC-MS. The amounts of FFA in FCS and BSA were very low and can be ignored in relation to the FFA- and LysoPC-supplemented media; this was validated by gas chromatography. For the LysoPC-supplemented media a concentration of 450 μM of the respective LysoPC species was added to the BSA containing medium. As LysoPC has good (micellar) water solubility, a stock solution of LysoPC in PBS was prepared at a concentration of 180 mM and dissolved in BSA containing medium to obtain the final concentration of 450 μM. Long chain fatty acids have poor water solubility and were first dissolved in EtOH at 56 °C and at a concentration of 90 mM. The resulting clear solution was very slowly added to the BSA containing medium under intensive stirring. LysoPC removal of LysoPC 17:0 (1-heptadecanoyl-2-hydroxy-*sn*-glycero-3-phosphocholine), LysoPC 18:0 (1-stearoyl-2-hydroxy-*sn*-glycero-3-phosphocholine), and LysoPC 18:1 (1-oleoyl-2-hydroxy-*sn*-glycero-3-phosphocholine; all obtained from Avanti Polar Lipids, USA) were investigated using 2 × 10^5^ solid epithelial tumour cells and 1 × 10^6^ leukaemia tumour cells, respectively, cultivated in 24-well tissue culture plates with 1 ml medium, either BSA medium as control, or 450 μM LysoPC supplemented medium. Cells and supernatants were separated after 0, 24, 48, and 72 h of incubation, cell culture supernatants were collected from triplicates of LysoPC-treated and untreated control cells. Cells were removed using 0.25 % trypsin/EDTA. Centrifuged supernatants and washed cell pellets were stored at −20 °C and −80 °C until further analysis, respectively.

For the incubation of B16.F10 cells (2 × 10^5^) with two different LysoPC species, a mixture of LysoPC 18:0 and 18:1 (450 μM) was simultaneously added (100/0; 80/20; 60/40; 40/60; 20/80 and 0/100; mol%/mol%). Cells and supernatants were separated after 48 h of incubation and cell pellets were analysed with regard to their FA composition change.

For the comparison of LysoPC and FFA incorporation into cellular lipids, cells (B16.F10, MV3 and AsPC1) were grown in 24-well tissue plates (2 × 10^5^/well) in 1 ml 450 μM LysoPC medium (LysoPC 17:0, 18:0, and 18:1) or the respective 450 μM FFA-medium (FFA margaric acid (C17:0), stearic acid (C18:0) and oleic acid (C18:1); all from Sigma Aldrich, Germany). After 0, 24, 48, and 72 h, cells were harvested and cellular lipids analysed using gas chromatography.

Cell proliferation was investigated using a BrdU-assay (Roche diagnostics GmbH, Germany) with cells grown in 96-well plates in 200 μl medium for 48 h. Adherent cells were labelled and the BrdU-assay was performed according to the manufacturer’s instructions.

### Determination of LysoPC and FFA concentration in supernatants

LysoPC concentrations in supernatants were determined after cultivating 2 × 10^5^ cells with LysoPC (450 μM) for 0, 24, 48, and 72 h, and separating cells and supernatants. The LysoPC concentration was determined by an enzymatic PL (PC/LysoPC) assay containing phospholipase D and choline oxidase (mti diagnostics GmbH, Germany). For the specific determination of free choline, a similar assay without containing phospholipase D was developed. Glycero-3-phosphocholine (GlyceroPC) was determined using the assay for free choline adding 1 IU/mL of sn-glycerol-3-phosphocholine phosphodiesterase (Sigma). We ensured the specificity of the three assays by analysing different concentrations of LysoPC, free choline, GlyceroPC, and PC.

For the quantification of FFA in supernatants, FA-free BSA had to be used. Supernatants of B16.F10 cells were incubated with LysoPC 17:0 and 18:1 (450 μM), respectively, for 0, 24, 48, 72, and 144 h. FFA concentration was determined using a FFA assay (Roche diagnostics GmbH, Germany) according to the manufacturer’s instructions.

The degradation of LysoPC in cell-free supernatant after pre-incubation with B16.F10 cells was determined by incubating cells grown confluent in 24-well plates with 1 ml LysoPC 17:0 for 6 h, followed by separation of the supernatant and subsequent cell-free incubation for 0, 24, 48, 72, and 96 h. As control experiments, LysoPC 17:0 medium without cells and LysoPC 17:0 with cells were accordingly incubated and processed.

### Determination of cellular FA composition (total, neutral lipid and PL)

Total lipid from harvested and frozen cell pellets (2 × 10^6^) after 0, 24, 48, and 72 h of incubation with either BSA, LysoPC, or FFA supplemented medium, were isolated by lipid extraction according to a modified method of Bligh and Dyer [46]. Neutral lipids and PL were separated using solid phase extraction as previously described [13, 47]. FA analysis of either total FA, or of the neutral lipids and PL-fraction separately, was performed by using a Gas Chromatograph HP-5890 Series II Plus analyser with flame ionisation detector. The settings were used as described previously [13], it was possible to identify changes in FA-pattern – total FA changes as well as the neutral lipids and PL-fraction separately – of tumour cells induced by the certain LysoPC or FFA-treatment.

### Confocal laser scanning microscopy

For the visualisation of lipid droplets (LD), B16.F10 cells were grown sub-confluent on sterile glass-cover-slips, either in LysoPC 17:0 medium (450 μM), or as a control in BSA medium. Following fixation with 4 % paraformaldehyde, cells were washed with PBS. Nuclei were stained with DAPI (Life Technologies GmbH, Germany), LD were stained with BODIPY 493/503 (Life Technologies GmbH, Germany) 1:500 dilution in 0.9 % NaCl of the stock solution (1 mg/ml in ethanol) for 10 min followed by a washing step. The glass cover slips were attached to a cover glass with mounting medium (MobiGlow, MoBiTec GmbH, Germany) and the samples were then analysed in a confocal laser scanning electron microscope LSM 510 Meta with 20 x/1.4 NA objective lens (Zeiss, Germany). Three to four pictures were taken from each sample and then processed using the Zen2009 software programme.

### Fluorescence recovery after photobleaching (FRAP)

5 × 10^3^ B16.F10 cells were seeded on μ-dishes (ibidi, Germany) previously coated with 10 μg/ml fibronectin (Roche Diagnostics GmbH, Germany) and incubated with LysoPC containing medium for 72 h. Medium was removed and cells were washed with PBS and incubated with 4 μmol/L 18:1 NBD-PE (1,2-dioleoyl-sn-glycero-3-phosphoethanolamine-N-(7-nitro-2-1,3-benzoxadiazol-4-yl)) (ammonium salt) (Avanti Polar Lipids, USA) solution in PBS with 2 % ethanol for 10 min at 37 °C to achieve insertion into the membrane. After washing once with PBS, bleaching was performed with a 488 nm laser, used at a maximum intensity for 280 ms. Fluorescence recovery was measured for 60 s at 0.6 % laser intensity with a Nikon A1 confocal microscope. Kinetics of fluorescence recovery were calculated according to Ishikawa-Ankerhold et al. and shown as half-life of fluorescence recovery [48].

### Cell migration

B16.F10 cells (1 × 10^5^) were seeded into each well of a 24 well plate (Greiner Bio-One, Germany), partially coated with 10 μg/ml collagen (Roche Diagnostics GmbH, Germany), and incubated with LysoPC. After 72 h, a scratch wound was induced into the confluent cell monolayer by a pipette tip. Wound healing was observed for 12 h at 37 °C and the speed of migration was determined by linear regression.

### Animal experiments

All animal experiments were performed in accordance with the German Animal License Regulations (Tierschutzgesetz) identical to UKCCCR Guidelines for the welfare of animals in experimental neoplasia [49]. Male C57Bl/6 mice were obtained from Charles River (Sulzfeld, Germany) at an age of 8 to 12 weeks.

### Injection of tumour cells, detection of metastatic spread

To induce lung metastases, 2 × 10^5^ B16.F10 melanoma cells in 100 μl PBS were intravenously injected into the tail vein of each mouse. The B16.F10 cells used were luciferase-transduced as previously described [16]. To investigate the effects of the pre-treatment of tumour cells with different lipid containing media on their metastatic behaviour, each of the 10 mice received B16.F10 cells pre-treated for 10 days with 450 μM LysoPC 18:0, LysoPC 18:1, FFA C18:0, or FFA C18:1; BSA treated cells were used as control. Experiments were terminated on day 18 after injection of tumour cells, lungs were removed and weighted, and stored as snap-frozen samples. Metastatic lesions were quantified by homogenising the mice’s lungs in luciferase lysis buffer (Promega, Germany) and measured in a luciferase assay as previously described [50].

### Plasma LysoPC levels

For blood collection, mice were anaesthetised with isoflurane (2.5 %, 3 l/min O_2_) and samples were collected carefully from the retro-orbital plexus by using glass capillaries in heparinised tubes. Blood samples were centrifuged at 2320 × g for 5 min and plasma was stored at −80 °C until analysis. For the lipid extraction, 440 μl PBS was pipetted into 15 ml glass tubes, frozen plasma samples were thawed and 20 μl was added to the PBS. 20 μl of LysoPC 19:0 (100 μM) were added to each sample as internal standards. Extraction was performed as described by Zhao et al. [10] with three repeated extraction steps. The combined solvents were evaporated under a nitrogen stream at 40 °C until complete dryness. Prior to analysis, the dried lipid extracts were dissolved in 100 μl methanol.

LysoPC-analysis was carried out using a Quadrupol API 2000 MS/MS mass spectrometer (AB Sciex, Germany), equipped with an Agilent 1100 LC system (Agilent Technologies, USA). Acetonitrile was used as mobile phase A, and H_2_O with 10 mM ammonium acetate, pH 8 was used as mobile phase B with a Waters XBridge BEH HILIC Column, 130 Å, 3.5 μm, 3 mm × 150 mm analytical column (Waters, USA). The LC gradient started with 90 % A from 0 to 0.1 min, down to 76.5 % A at 22.5 min, returning from 76.5 % A to 90 % A at 22.6 min and held at 90 % A until 27.5 min. Flow rate was 0.5 ml/min and column temperature was maintained at 50 °C throughout the analysis. Parameters for the analysis in the positive ion mode with the TurboIonSpray® source were: source temperature: 400 °C, capillary voltage: 5500 V, desolvation gas 20 l/h, focusing potential: 380 V, declustering potential: 35 V, collision energy: 40 V. The nitrogen required as collision and curtain gas was produced by an NGM 11-LC-MS nitrogen generator. 10 μl of each sample was injected by the Agilent 1100 LC systems auto sampler.

Quantitative analysis was performed in the multiple reaction monitoring (MRM) mode, monitored ions were at m/z 468.3 (parent ion)–184 (product ion) for LysoPC 14:0; 496.0-184 for LysoPC 16:0; 510.3-184 for LysoPC 17:0; 524.0-184 for LysoPC 18:0; 522.0.3-184 for LysoPC 18:1; 520.0-184 for LysoPC 18:2; 518.3-184 for LysoPC 18:3; 538.4-184 for LysoPC 19:0; 544.0-184 for LysoPC 20:4; 542.3-184 for LysoPC 20:5; 568.0-184 for LysoPC 22:6 and 608.5-184 for LysoPC 24:0. LysoPC standard calibration curves were established for quantitative analyses and were performed for each analysis batch. Therefore plasma samples were spiked with known concentrations of LysoPC 19:0 (1, 10, 50, 100, 250, and 500 μM). A standard curve was derived which served as calculation template for the analysed LysoPC. The peak area of each analyte was integrated and results were calculated using the API 2000 software and a self-programmed Microsoft-EXCEL evaluation template.

### Manipulation of LysoPC plasma levels and effects on metastatic spreading due to different supplementations

Standard laboratory chow was supplemented with 2 % hydrogenated egg-phosphatidylcholine (EPC3; Lipoid GmbH, Germany) and was purchased from Ssniff Spezialdiäten GmbH, Germany. The experiment included five treatment groups with five animals per group. Group 1 (healthy mice) served as control group, these animals received EPC3-supplemented chow and no tumour cells. In the PC feeding groups, mice received EPC3-supplemented chow administered either as prophylaxis, starting one month prior to tumour cell injection (group 2 – “EPC3_pro_”) or as therapy, starting after tumour cell injection (group 3 – “EPC3_ther_”), the latter mice were kept on a normal diet until the tumour cells were injected, and were set onto the EPC3-enriched diet one day after injection until the end of the experiment. During the whole study, mice had free access to feed chow. For the LysoPC s.c. treatment (group 4), animals were kept on a normal diet and were subcutaneously injected with LysoPC 17:0 at a dose of 20 mg/kg in PBS containing 2 % BSA. Injections were given six times at 12 h intervals, beginning 23 h before tumour cell injection. The “tumour growth control group” (group 5) received the standard laboratory chow without EPC3-supplement and served as a control for the growth of tumour cells, which were injected simultaneously with the other groups. Mice were supervised for weight progression, food intake and general behaviour daily.

### Liposome injection

Liposomes were manufactured by dual centrifugation as previously described [51] and consisted of hydrogenated DSPC (1,2-distearoyl-*sn*-glycero-3-phosphocholine; CordenPharma, Switzerland) / cholesterol (70:30 molar ratio; Sigma Aldrich, Germany). Before use, the liposomal gel was redispersed in steril NaCl-solution (0.9 %). Injection dose was 5 ml/kg bodyweight; liposomes were injected into tail veins of the mice. Blood samples were taken from anaesthetised mice 2 h after injection.

### Statistics

The statistical analysis was performed with GraphPad Prism 5.01. Analysis of differences was performed with Student’s t-test and ANOVA. The reported values are means from at least three identical experiments, unless otherwise stated. Significant differences were indicated at *p*-value < 0.05.

## Abbreviations

BSA: Bovine serum albumin
EPC3: Hydrogenated egg-phosphatidylcholine
FA: Fatty acid
FFA: Free fatty acid
GlyceroPC: Glycerophosphocholine
LD: Lipid droplets
LysoPC: Lysophosphatidylcholine
LysoPLA: Lysophospholipase A
MTD: Maximum tolerated dose
PC: Phosphatidylcholine
PL: Phospholipid
